# Improved genome sequencing using an engineered transposase

**DOI:** 10.1186/s12896-016-0326-1

**Published:** 2017-01-17

**Authors:** Amirali Kia, Christian Gloeckner, Trina Osothprarop, Niall Gormley, Erin Bomati, Michelle Stephenson, Igor Goryshin, Molly Min He

**Affiliations:** 1 Department of Protein Engineering, Illumina Inc, 5200 Illumina Way, San Diego, CA USA; 2 Technology Development, Illumina Inc, Little Chesterford, Nr Saffron Walden, Essex CB10 1XL UK; 3 NEO New Oncology GmbH, Gottfried-Hagen-Str. 20, Cologne, 51105 Germany; 4 Illumina Inc, 5602 Research Park Blvd., Suite 200, Madison, WI USA

## Abstract

**Background:**

Next-generation sequencing (NGS) has transformed genomic research by reducing turnaround time and cost. However, no major breakthrough has been made in the upstream library preparation methods until the transposase-based Nextera method was invented. Nextera combines DNA fragmentation and barcoding in a single tube reaction and therefore enables a very fast workflow to sequencing-ready DNA libraries within a couple of hours. When compared to the traditional ligation-based methods, transposed-based Nextera has a slight insertion bias.

**Results:**

Here we present the discovery of a mutant transposase (Tn5-059) with a lowered GC insertion bias through protein engineering. We demonstrate Tn5-059 reduces AT dropout and increases uniformity of genome coverage in both bacterial genomes and human genome. We also observe higher library diversity generated by Tn5-059 when compared to Nextera v2 for human exomes, which leads to less sequencing and lower cost per genome. In addition, when used for human exomes, Tn5-059 delivers consistent library insert size over a range of input DNA, allowing up to a tenfold variance from the 50 ng input recommendation.

**Conclusions:**

Enhanced DNA input tolerance of Tn5-059 can translate to flexibility and robustness of workflow. DNA input tolerance together with superior uniformity of coverage and lower AT dropouts extend the applications of transposase based library preps. We discuss possible mechanisms of improvements in Tn5-059, and potential advantages of using the new mutant in varieties of applications including microbiome sequencing and chromatin profiling.

**Electronic supplementary material:**

The online version of this article (doi:10.1186/s12896-016-0326-1) contains supplementary material, which is available to authorized users.

## Background

Library construction plays an important role for high-throughput next generation sequencing (NGS). Plethora of library construction methods have been developed in the past few years [1–3]. Most common library preparation methods follow a basic procedure with minor variations [1]. This procedure is usually lengthy and includes several steps. First, DNA is fragmented by sonication, nebulization or shearing to desired sizes. The fragmented DNA is then repaired and end-polished including blunt-end and A-tailing. Finally, platform-specific adaptors are ligated to DNA library [2, 4]. Typically, this process results in significant sample loss, and therefore, requires a DNA input amount of over 200 ng, sometimes up to 1 ug. This workflow also limits the throughput. Nextera, developed by Epicentre (an Illumina company), is an alternative approach to streamline the workflow, improve turnaround time and reduce DNA input and increase throughput. Nextera takes advantages of an in vitro transposition reaction, using a transposase Tn5 and a free transposon end that contains a transposase recognition site Mosaic End (ME) and the sequencing adaptor to form a Transposome™ complex. When this complex is incubated with target double-stranded DNA (dsDNA), the target is fragmented and the transferred strand of the transposon end including the sequencing adaptor is covalently attached to the 5′ end of the target fragment, resulting in a sequenceable DNA library [5, 6]. Combining DNA fragmentation and sequencing adaptor tagging in one single tube reaction results in fast turnaround time of less than 2 h and requires low DNA input as little as 50 ng. In addition, by varying the concentration of Transposome complexes relative to the input DNA, the size distribution of the fragmented and tagged DNA library can be controlled. Nextera libraries can also incorporate barcodes, enabling multiplexed sequencing on a single instrument run, and thus significant cost savings.

The transposase in the Transposome complex integrates the ME into target sites by joining its 3’OH termini to staggered positions on the top and bottom DNA strands of the target. This staggered joining results in a target site duplication of a defined number of base pairs, which can be used to map precisely the site of integration for the transposon [7, 8]. In genome sequencing applications, it is important that the Transposome inserts the sequencing tags into target DNA with little to no sequence bias, leading to more uniform and complete coverage of insertion sites. By using in vitro analysis of the insertion sites of a fosmid model system [9, 10], it has been reported that many transposases have preferred insertion sequence bias. For example, Tn5, Mu and Hermes bias towards the sequences containing guanosine (G) and cytidine (C) [9, 11], while Mos1 and PiggyBac bias towards the sequences containing adenosine (A) and thymine (T) [12–14]. Although ligation based methods have their own bias [15], the transposases’ insertion bias is usually more pronounced. This sequence preference yields less uniform spacing of insertions and hence potentially less uniform coverage in genome sequencing.

In this study, we engineer Tn5 transposase to have altered insertion bias. The changed insertion bias in mutant Tn5-059 with mutations K212R/P214R/G251R/A338V, leads to more uniform coverage of a bacterial genome *B. cereus.* We further demonstrate the benefits of using Tn5-059 in human genome sequencing including more uniform coverage and less sensitivity to DNA input amount variation. We discuss the mechanism of these improvements, strategies to further improve coverage uniformity, and new applications that could take advantage of these features of engineered Tn5-059.

## Methods

### Tn5 mutant library construction

Tn5 mutants were generated by random mutagenesis using error prone PCR on the entire wild type Tn5 transposase (NCBI accession code ‘3ECP_A’, Additional file 1). Site saturation was performed on the modeled DNA binding site of the protein. The mutagenized Tn5 fragments were inserted into a modified pET11a vector for expression in *E. coli* (Illumina Madison). The vector is kanamysin resistant for plasmid stability and has a Strep Tag II-sumo fusion downstream of the T7 promoter/lac operon at the N-terminus of Tn5 coding region to aid purification. The driver mutations identified by linear regression were combined by standard site-directed mutagenesis (Qiagen).

### Mutant protein expression and purification

Mutant library was plated, single colonies were selected to inoculate 1 L Luria Broth (LB) media with 50 μg/mL kan and were allowed to grow to OD600 = 0.5. Expression of Tn5 mutant transposases was then induced by addition of 100 μM Isopropyl β-D-1-thiogalactopyranoside (IPTG) and continued incubation at 18 °C for 19 h.

Cells were harvested by centrifugation and resuspended in TNE1 buffer (100 mM Tris, pH 8.0, 1 M NaCl, 1 mM Ethylenediaminetetraacetic acid (EDTA), 1 mM Dithiothreitol (DTT)) containing complete protease-inhibitor mix (Roche). A glass homogenizer was used to break-up the cell pellet before being passed through a microfluidizer three times for lysis. Sodium deoxycholate was added to the lysate (0.1% final) and the mixture was incubated at room temperature while stirring for 15 min followed by 15 min at 4 °C. While stirring at 4 °C, 5% polyethylenimine, pH 7.5 was added to the mixture (0.5% final) and stirred further for 1 h to precipitate nucleic acids which was removed by centrifugation (45,000 g for 20 min at 4 °C). Saturated ammonium sulfate was added to the supernatant at a 1:1 ratio and the mixture was stirred at 4 °C for 1 h and then centrifuged (45,000 g for 20 min at 4 °C). The pellet containing the Tn5 mutant proteins was resuspended in 10 mL TNE1, centrifuged to remove particulates and the resulting supernatant was further diluted 5× with TNE1 and loaded onto a StrepTrap High Performance (HP) column (GE Healthcare) that was equilibrated with TNE1 using an AKTA Pure (GE Healthcare).

Post load, the StrepTrap HP column was washed with 10 column volumes (CV) of 100 mM Tris, pH 8.0, 4 M NaCl, 1 mM EDTA followed by 10 CV 100 mM Tris, pH 7.5, 100 mM NaCl, 1 mM EDTA. The protein was then eluted with a 10 CV gradient using 100 mM Tris, pH 7.5, 100 mM NaCl, 1 mM EDTA, 5 mM Desthiobiotin (IBA-lifesciences). Fractions containing peaks at OD280 were pooled and applied to a HiTrap Heparin HP column that was equilibrated with 100 mM Tris, pH 7.5, 100 mM NaCl, 0.2 mM EDTA (GE Healthcare). After binding, the column was washed with 15 CV equilibration buffer followed by a 20 CV salt gradient (100 mM-1 M NaCl). A single eluted peak at 0.5 M NaCl was shown by sodium dodecyl sulfate polyacrylamide gel electrophoresis (SDS-PAGE) to contain the Strep-Sumo-Tn5 mutants at 66 kDa. The eluted peak was concentrated in a Vivaspin 20 centrifugal concentrator with a 10 kDa MWCO then diluted 1:1 with 100% glycerol for storage at −20 °C. From this expression and purification, the yield of Tn5 mutant transposases was approximately 5 mg per 1 L culture.

### Transposome assembly and activity normalization

The 19 bp Tn5 mosaic end (ME) transposon sequence (also containing the 14 and 15 bp 5’ adaptor sequence compatible with Illumina paired-end sequencing) was annealed by heating the single stranded oligos at 95 °C for 5 min then reducing the temperature 5 °C every 2 min down to 20 °C. The annealed ME’s were combined with purified Tn5 transposases at a 1.2:1 molar ratio (ME:transposase) and incubated at 37 °C for 1 h. The resulting Transposome assembly was stored at −20 °C until use.

Tagmentation activity of each mutant was normalized using the standard method and reagents specified in the Nextera DNA library preparation method (Illumina). In short, 25 ng *B. cereus* genomic DNA (gDNA) was tagmented by various concentrations of mutants or standard Tn5 from Illumina Nextera kit as a control. The size of resulting fragmented DNA was analyzed on Agilent’s High Sensitivity DNA bioanalyzer chip. The concentrations of Tn5 mutants were then normalized to achieve the same DNA fragment size distribution where the area under the curve between 100 and 300 bp was 20–30%, 301–600 bp 30–40%, and 601–7000 bp 30–40%, whereas the total area under the curve between 100 and 7000 bp was ≥90% (Additional file 2: Figure S1).

### DNA library preparation, sequencing and data analysis

5 μL of each Tn5 mutant Transposome at normalized concentration was used to prepare sequencing libraries for *E. coli* gDNA (balanced genome), *R. sphaeroides* (GC rich genome), and *B. cereus* genomic DNA (AT rich genome) using the standard Nextera DNA library preparation method. The libraries were then sequenced on MiSeq following Illumina’s standard protocol. Bias plots was generated by counting the number of times each nucleotide was observed in each cycle for all the reads and reporting it as a percentage. Bias plot shows an overall tagmentation bias of a transposase. Note that the bias at each position can be independent of other positions. Coverage plots show the percentage of bases observed at different sequencing depth. They were generated using samtools’ mpileup option (http://www.htslib.org/). Normalized GC curves and AT/GC dropouts were generated using Picard Tools (http://broadinstitute.github.io/picard/).

### Linear regression

Linear regression models were used to estimate the weight of each individual mutation in the insertion bias. Each linear regression model was applied to the content of the dominant nucleotide at a base position in the reads, resulting in a model per base position. *E. coli* sequencing results were used for fitting the models. Hence, the following nucleotides where used as the dominant nucleotide between base positions 1 and 15: GTTTA***CTGTGCG. Since Tn5 acts as a homodimer, dominant nucleotides observed at positions 6 through 9 are always complement bases to those in positions 1 through 4. Therefore, we ignored models for bases 6, 7 and 8 but kept base position 9. Although position 9 replicates the behavior of position 1 due to symmetry, position 1 is affected by sequencing artifacts so we use position 9 to better capture the features of position 1.

Ten-fold cross validation was used for training and weights were averaged through the 10 cross trainings. Input matrix for the predictor variables consisted of rows for each mutant and columns for all observed mutations. Mutations that always appeared together caused singularity in the matrix. Hence, all but one of the columns associated with those mutations were dropped. Least square method was used to solve for the weight vectors.

For each position, the mutations that had significant positive or negative weights were picked. New mutant library was created by combining mutations from different positions. Hence, mutations are grouped based on similar effect. Groups may have common mutations that have effect on multiple positions simultaneously.

### Tn5/DNA binding stability assay

Standard Tn5 was shown to remain bound to its target DNA post tagmentation (unpublished results). Therefore, gap fill of the tagmented DNA by a polymerase and further amplification of the DNA by PCR will be prevented by steric hindrance. However, Tn5 dissociates from the tagmented DNA upon elevated temperature, thus allowing further gap fill and PCR of the DNA by a polymerase. The temperature required to allow PCR reaction of a polymerase can thus be used to compare Tn5/DNA binding stabilities of various Tn5 mutants. The higher temperature required, the more stable the complex.

The mutant Tn5-059 or standard Tn5 was used to tagment *B. cereus* gDNA in 1 mL reaction by following the standard Nextera protocol except the TD buffer was replaced by 20 mM Tris Acetate pH 7.5, 5 mM magnesium acetate. TD buffer contains magnesium, so this should not create an extra combined effect with the mutations to change the insertion bias. Aliquots of 25 μL reaction were dispensed into a PCR plate in triplicate and were incubated at 55 °C for 5 min followed by centrifugation (1000 g for 5 min at 4 °C).

For the second step containing PCR, a PCR mix was prepared by combining 200 μL PPC (PCR Primer cocktail), 200 μL i501, 200 μL i507, and 400 μL NPM (reagents supplied in the standard Nextera DNA library preparation kit). 25 μL of this mix was then added to each of the 25 μL tagmentation reaction aliquot on the PCR plate, mixed by pipette and returned to the thermocycler. The temperature gradient between 72 °C and 95 °C was generated across the 12 columns of the plate and held for 1 min, the plate was then incubated at 72 °C for 5 min followed by 98 °C for 30 s. After this gap fill-denaturation step, 5 cycles of PCR specified in the Nextera DNA library preparation method was performed. The plate was then centrifuged at 1000 g for 5 min at 4 °C. Each tagmentation-PCR amplified reaction was then purified using a Zymo Clean and Concentrator 96 well plate and eluted with 25 μL 10 mM Tris, pH 8.0. A triplicate of negative control sample was prepared following the standard tagmentation protocol including Zymo cleaning to remove Tn5 transpoase but without the PCR step. A triplicate of positive control was prepared following the standard tagmentation protocol including Zymo cleaning to remove Tn5 transposase and the PCR step to amplify tagmented DNA. All purified DNA products were then diluted 1:10 in 10 mM Tris, pH 8.0 and quantitated using Picogreen and a lambda DNA standard.

The results demonstrated that standard Tn5 releases from the tagmented DNA and thus allows subsequent gap fill and PCR reactions at 74.2 °C, while Tn5-059 does so at 76.6 °C (Additional file 3: Figure S2). The higher temperature required for Tn5-059 indicated a more stable DNA binding complex.

## Results and Discussions

**Fig. 1:**
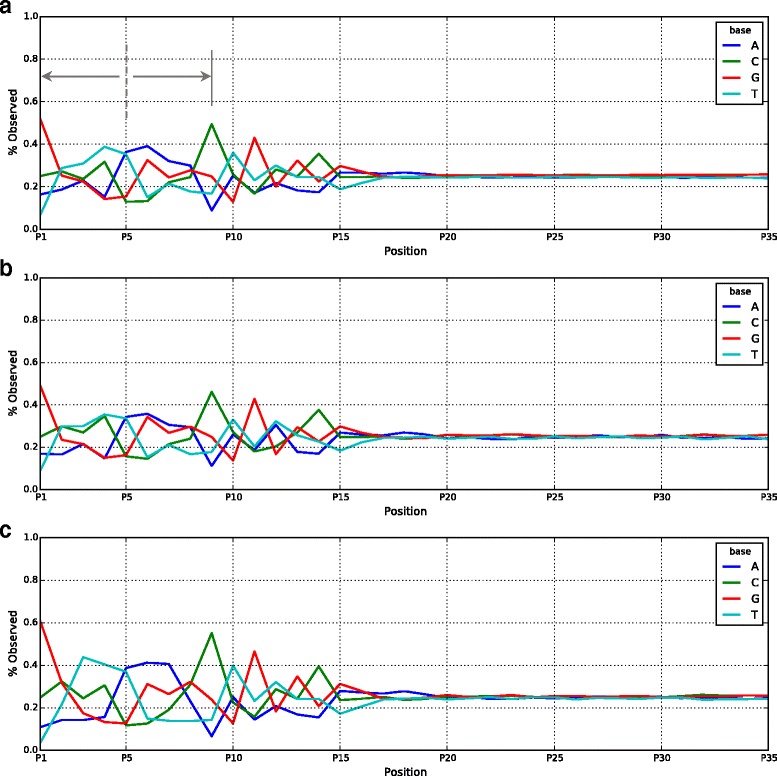
Bias *plots*, showing percentage of observed bases at each position (or sequencing cycle). *Plots* show insertion bias for standard Tn5 in NexteraV2 kit and two Tn5 mutants from sequencing *E. coli* genomic DNA. The intensities after position (cycle) 20 are the base composition of *E. coli* genomic DNA. **a** Standard Tn5 in NexteraV2 kit. Notice the symmetry in the *plot* centered at position 5, between positions 1 through 9 **b** Tn5 mutant, notice the change in the insertion bias at positions 3 through 7 and position 12 **c** Tn5 mutant, notice the change in the insertion bias at positions 3 through 7, as well as higher G bias at position 1

Random mutagenesis library of Tn5 transposase that aims to cover the entire coding region is constructed such that every mutant has approximately 1 to 6 random mutations. About 1000 such mutants are expressed, purified and used in a standard Nextera protocol to tagment *E. coli* genomic DNA followed by sequencing analysis of the DNA libraries. Bias plots (Fig. 1) are used to assess the insertion bias of a transposase by comparing the percentage of observed bases at each base position (or sequencing cycle) to the average base composition of *E. coli* genome. Standard Tn5 has an insertion bias of 21 bases, 15 of which can be observed in a sequenced read (Fig. 1a). The bias plot demonstrates a symmetry between base 1 and base 9 with the center of the symmetry at base 5 (Fig. 1a). Since Tn5 functions as a homodimer [7, 8], we hypothesize that the 9 base pair overlap of the target DNA within the two monomers results in this symmetry (Additional file 4: Figure S3). Out of the 1000 mutants we examined, many of them showed altered bias plots (two examples are shown in Fig. 1b and c), indicating their insertion bias have changed at some base positions, likely caused by the positions of mutations.

**Fig. 2:**
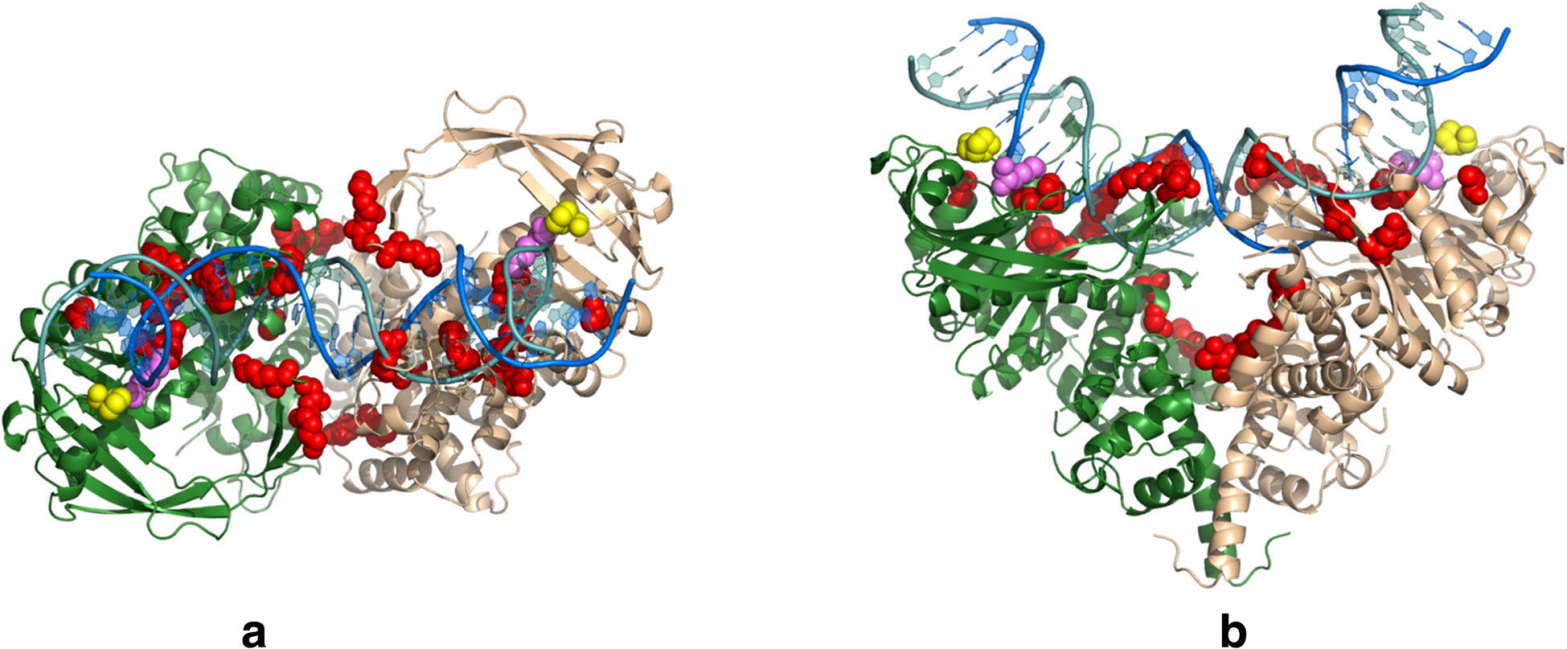
Tn5 dimer with selected driver amino acids in spherical representation. These positions are selected based on multiple linear regression models. K212 is in *pink* and P214 in *yellow*. **a** Top view. **b** Side view

Since every mutant in the random library usually contains more than one mutation, we use linear regression models to segregate the effect of each mutation in a mutant, and subsequently to identify all the driver mutations that cause changes in insertion bias. A separate linear regression is applied at each base position that contains an insertion bias. This led to the extraction of the individual effects of each mutation on the insertion bias at different base positions (See Additional file 5: Figure S4 and Additional file 6: Figure S5). Not surprisingly, most driver mutations are found close to the target DNA binding site or at the interface of two Tn5 monomers (Fig. 2). We then combine the driver mutations for each base position to create a set of combination mutants (Table 1).

**Table 1:** Selected positions from linear regression for further recombination and constructing super mutants. Column headers indicate the nucleotide position starting from where Tn5 inserts its ME (Also refer to Fig. 1 and Additional File 4: Figure S3)

P1	P2	P3	P4	P5	P9	P10	P11	P12	P13	P14
	I046	T047	I046	W125	A195	A338	E146	K212	P214	
	R138	D119	G251	H127	M343		E190			
	T334	K249	S264							
	W125	K252	A338							
	K249									

**Fig. 3:**
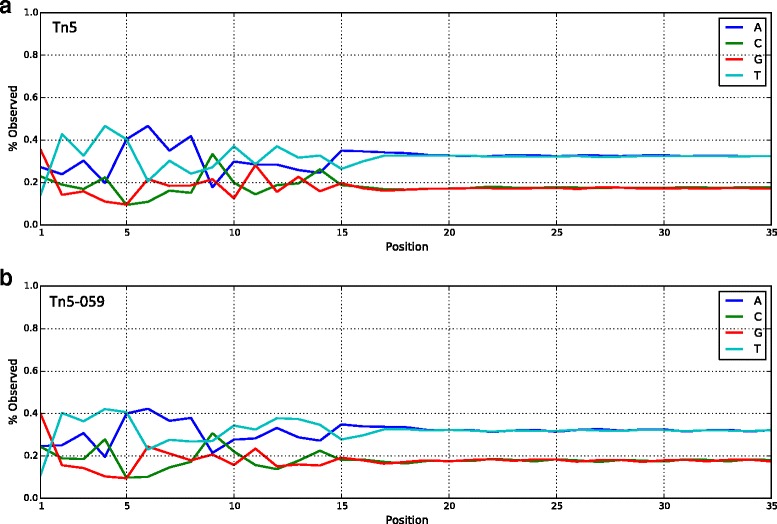
Percentage of observed bases at each cycle using standard Tn5 in NexteraV2 kit (**a**), or Tn5-059 (**b**) for sequencing *B. cereus* genomic DNA. In particular, the two *plots* differ at positions 3, 4, 6, 7, 11, 13 and 14. For Tn5-059, the bias within positions 10–15 is much closer to the overall genome composition

The combination mutants are then expressed, purified and used to prepare sequencing library from *B. cereus* genomic DNA. We selected B. *cereus* genomic DNA at this stage since it has higher AT content and Tn5 is known to have a GC insertion bias. B. *cereus* will help us stress our selection assay and hence better differentiation of the performance of mutants. Figure 3 shows the difference in insertion bias between NexteraV2 and mutant Tn5-059 (K212R/P214R/G251R/A338V). Interestingly, we observe a structural correlation between the positions of the mutations and the positions of the DNA bases that displayed changes in the bias plot. For example, mutations K212R/P214R in Tn5-059 are in close proximity to bases 10–15 (Fig. 2a) in the structural model.

The observed bases between positions 10 and 15 are better separated in bias plot and are closer to the overall genome composition, indicating less insertion bias (Fig. 3b). New mutations can also create artificial distortions to the bias plot and alter the expected symmetry in the plot. For example, a single mutation at K120, which is located in the middle of the dimer complex and most probably interacts with the target DNA, can cause the target DNA overlap to alternate between 9 bp and 10 bp or to switch completely to 10 bp (Additional file 7: Figure S6). These changes in target DNA overlap will distort the symmetry in the bias plot, which in turn have a spurious effect in the linear regression models, forcing it to pick these mutations as false positive mutations. The actual effect of these mutations on activity remains unknown and needs further investigation.

**Fig. 4:**
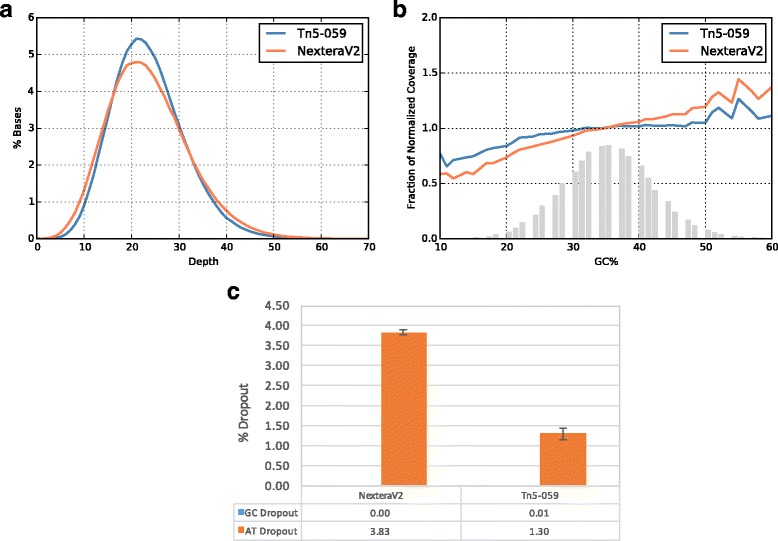
Sequencing results for *B. cereus* genomic DNA. Libraries are prepared using 50 ng of input DNA. **a** Uniformity of Coverage. Sequencing results are down sampled to 24× coverage. Tn5-059 shows improved uniformity when compared to standard Tn5. **b** Normalized GC *plot*. Grey bar shows schematic GC composition of *B. cereus* genome. Tn5-059 has a more uniform coverage with less AT dropout. **c** AT/GC dropout percentages. Both enzyme have no GC dropout while Tn5-059 shows significant improvement in AT dropout

We compare the full sequencing metrics of *B. cereus* genomic DNA prepared by Tn5-059 or standard Tn5 in NexteraV2 kit, respectively. Mutant Tn5-059 shows less under-covered and over-covered regions when compared to standard Tn5, confirming Tn5-059 yields more uniform coverage of *B. cereus* genome (Fig. 4a). Normalized GC plots, defined as the number of reads per window normalized to the average number of reads per window across the whole genome are shown in Fig. 4b. Tn5-059 has a more uniform normalized coverage over regions with different GC content than standard Tn5 (Fig. 4b), which is a result of improved AT dropout (Fig. 4c). In the case of *B. cereus genomics DNA, we observed approximately 65% reduction in AT dropout.* We conclude that the improvement observed in both coverage uniformity and AT dropout of Tn5-059 is a direct result of altered insertion bias (Fig. 3).

**Fig. 5:**
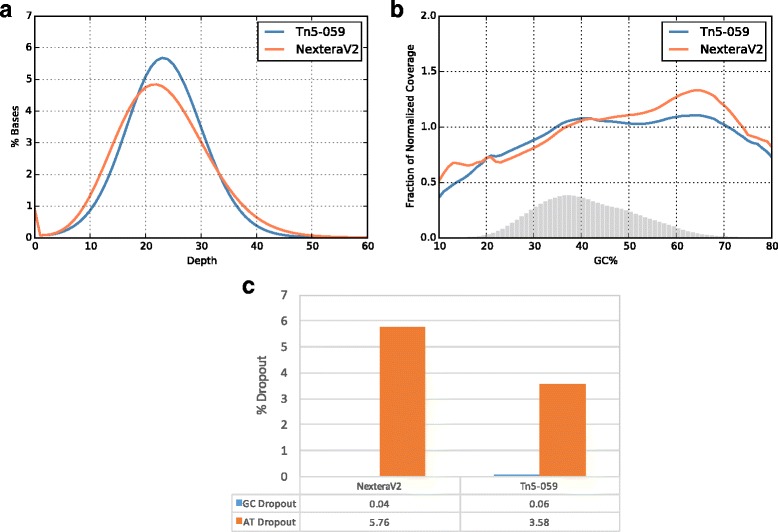
Sequencing results for Human genomic DNA. Libraries are prepared using 50 ng of input DNA. Similar enzyme concentrations of Tn5-059 and standard Tn5 are used and 2x151 bp sequencing run is performed on a HiSeqX. **a** Uniformity of Coverage. Sequencing results are down sampled to 20× coverage. Tn5-059 shows improved uniformity over standard Tn5 in NexteraV2 kit. **b** Normalized GC plot. Grey bar shows schematic GC composition of Human genome. Standard Tn5 has a clear bias towards GC rich regions while undercovers AT rich regions. **c** AT/GC dropout. Tn5-059 improves AT dropout while adding little on GC dropout

We further characterize the behaviors of Tn5-059 in human genome sequencing. In this experiment, activities of Tn5-059 and standard Tn5 are normalized. Similarly, a more uniform coverage is observed with Tn5-059 (Fig. 5a), resulted from approximately 35% reduction in AT dropout without significantly changing GC dropout (Fig. 5c). Consistently, we observe a more uniform normalized coverage, especially at regions with higher GC content, when compared to standard Tn5 (Fig. 5b). Another important metric for library preparation methods is library diversity, an estimate of number of unique molecules in the library generated [16]. Tn5-059 shows 20–50% higher library diversity, achieving an approximate ~64 million unique molecule for human exome sequencing, likely resulting from a more uniform coverage [17]. This feature allows less sequencing and thus lower cost to reach the same sequencing depth. It is interesting to observe that Tn5-059 forms a tighter complex with target DNA after tagmentation (Additional file 3: Figure S2). Mutations of Tn5-059 increased its positive charges in the DNA binding site. It is conceivable that tighter DNA binding of Tn5-059 increases its chance of insertion reactions with varieties of DNA motifs, and hence reduces its insertion bias. Tighter DNA binding of Tn5-059 also leads to increased efficiency of tagmenting limited amount of input DNA, as shown in chromatin accessibility studies [18].

**Fig. 6:**
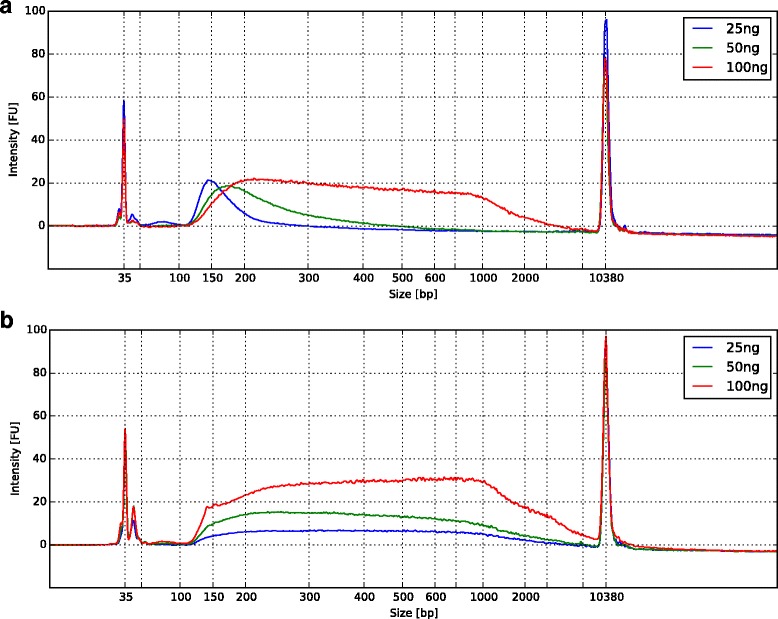
Fragment size distribution after tagmentation using Tn5 and Tn5-059. *Horizontal axis* is the fragment sizes in base pairs, *vertical axis* is the amount of observed fragments at different sizes in fluorescent units [FU] **a** Same concentration of Tn5 is applied to three different DNA inputs (25 ng, 50 ng or 100 ng). Lower DNA input shifts the fragment size distribution to the left, an indication of smaller fragments. **b** Tn5-059 demonstrates little changes in fragment size distribution over the same range of DNA input

Tn5-059 shows high tolerance to the input DNA for a large range of DNA input (Fig. 6). With the increasing amount of input DNA, the sizes of tagmented DNA increase for Tn5. Tn5-059, on the other hand, shows steady fragment size distributions over a wide range of DNA input amount. The hypothesis is that the lowered K_d_ of Tn5-059 to DNA reduces the ability of Tn5-059 to sample different DNA regions with various sequences, especially when transposase is in excess of DNA. If the catalytic efficiency remains the same as Tn5 or better, Tn5-059 is likely to insert into more varieties of sequences of DNA. Further characterizations of DNA binding and insertion kinetics of Tn5 vs. Tn5-059 is required to fully understand the mechanism.

## Conclusion

We have demonstrated that mutant Tn5-059 possesses the ability to tagment genomic DNA in a more uniform fashion that leads to better quality and lower costs of sequencing. In addition, Tn5-059 has higher DNA input tolerance and hence yielding a more robust system for low DNA input experiments. These features open up the utility of Nextera in genomic research. We can now enable Nextera-based library prep for microbiome sequencing, human whole genome sequencing and chromatin profiling even at the single cell level [18, 19].

## Additional files

Additional file 1: Starting sequencing for transposase Tn5. (TXT 608 bytes)
Additional file 2: Figure S1. Normalization of tagmentation activity based on pre-PCR insertion size distribution. Activity is normalized when 20–30% of the library has an insert size of 100–300 bp, 20–30% of 301–600 bp, and at least 90% of the library falls in the range of 100 bp-7000 bp. TDE1 refers to standard Tn5 Transposme complex that comes in Nextera kit. Here, TDE1 represents standard tagmentation using Nextera kit. (PDF 54 kb)
Additional file 3: Figure S2. DNA binding stability of Tn5 vs. Tn5-059. While heating at 74.2 °C is required for Tn5 to dissociate from tagmented DNA to allow following PCR amplification of DNA to reach the level of positive control, the temperature is elevated to 76.6 °C for Tn5-059 to do the same. The negative control is tagmentation without PCR amplification. The positive control is tagmentation followed by complete removal of transposase by Zymo cleaning to allow PCR amplification. (PDF 81 kb)
Additional file 4: Figure S3. Schematic representation of Tn5 tagmentation. There is 9 base pairs overlap between top and bottom strands. Two Tn5 transposase monomers, Tn5-A and Tn5-B form a dimer and tagment a double stranded DNA. Forward and reverse strands are shown in the figure. A typical bias plot from sequencing of *B. cereus* is also shown in the figure (refer to Fig. 3a). For every base at position P (where P is between 1 and 9) in a read, theoretically there is another read in the sequencing results that has complementary nucleotide to that base at position 10-P. This results in a symmetry between positions 1 and 9 in the bias plots, and the center of the symmetry will be at position 5. (PDF 113 kb)
Additional file 5: Figure S4. Linear regression weights on all mutated positions. In case of multiple mutations at a position, the one with the largest effect is shown. Negative values help decreasing the bias while positive values increase the bias. Each plot shows the results of a separate linear regression on a position in the bias curve. (PDF 300 kb)
Additional file 6: Figure S5. Linear regression weights on selected amino acids positions. This plot shows the different effect of a mutation on the insertion bias at different positions. (PDF 58 kb)
Additional file 7: Figure S6. Bias plots showing how bulky mutations at K120 distort or shift the symmetry of the graph. (a) NexteraV2 Tn5 (b) K120Y. Symmetry is distorted, resulting from a combination of 9 bp and 10 bp target DNA overlap, with 9 bp dominating (c) K120F. Similar to (b), but 10 bp overlap dominates (d) K120W. Complete switch to 10 bp overlap and the center of symmetry shifts from position 5 to middle of positions 5 and 6 (e) Top view of the Tn5 structure, MEs are shown in magenta color. Target DNA is schematically shown in the figure in shades of blue. K120 is shown in the spherical representation in color red (d) Top view of the structure. (PDF 1226 kb)

## Abbreviations

dsDNA: Double stranded DNA
DTT: Dithiothreitol
EDTA: Ethylenediaminetetraacetic acid
gDNA: Genomic DNA
HP: High Performance
IPTG: Isopropyl β-D-1-thiogalactopyranoside
LB: Luria Broth
ME: Mosaic End
NGS: Next-generation sequencing
SDS-PAGE: Sodium dodecyl sulfate polyacrylamide gel electrophoresis
