# Supplementary figures and images for: Improved genome sequencing using an engineered transposase
Source: BMC Biotechnol. 2017 Jan 17;17:6. doi: 10.1186/s12896-016-0326-1 (PMC5240201; doi:10.1186/s12896-016-0326-1)

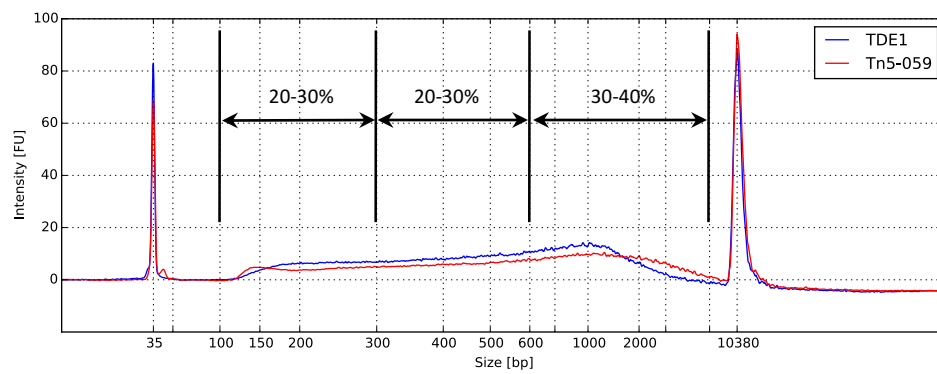

Supplement: Additional file 2: Figure S1. — Normalization of tagmentation activity based on pre-PCR insertion size distribution. Activity is normalized when 20–30% of the library has an insert size of 100–300 bp, 20–30% of 301–600 bp, and at least 90% of the library falls in the range of 100 bp-7000 bp. TDE1 refers to standard Tn5 Transposme complex that comes in Nextera kit. Here, TDE1 represents standard tagmentation using Nextera kit. (PDF 54 kb) [file 12896_2016_326_MOESM2_ESM.pdf]

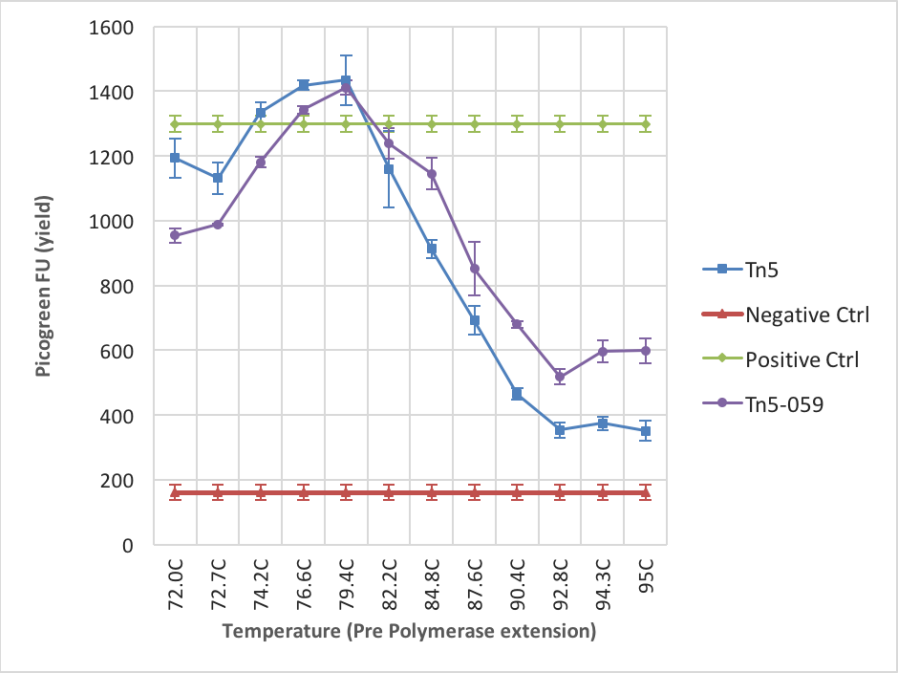

Supplement: Additional file 3: Figure S2. — DNA binding stability of Tn5 vs. Tn5-059. While heating at 74.2 °C is required for Tn5 to dissociate from tagmented DNA to allow following PCR amplification of DNA to reach the level of positive control, the temperature is elevated to 76.6 °C for Tn5-059 to do the same. The negative control is tagmentation without PCR amplification. The positive control is tagmentation followed by complete removal of transposase by Zymo cleaning to allow PCR amplification. (PDF 81 kb) [file 12896_2016_326_MOESM3_ESM.pdf]

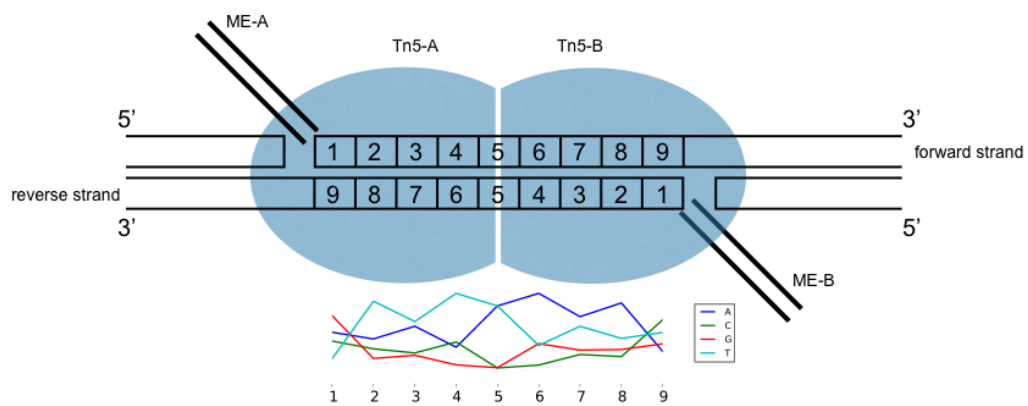

Supplement: Additional file 4: Figure S3. — Schematic representation of Tn5 tagmentation. There is 9 base pairs overlap between top and bottom strands. Two Tn5 transposase monomers, Tn5-A and Tn5-B form a dimer and tagment a double stranded DNA. Forward and reverse strands are shown in the figure. A typical bias plot from sequencing of B. cereus is also shown in the figure (refer to Fig. 3a). For every base at position P (where P is between 1 and 9) in a read, theoretically there is another read in the sequencing results that has complementary nucleotide to that base at position 10-P. This results in a symmetry between positions 1 and 9 in the bias plots, and the center of the symmetry will be at position 5. (PDF 113 kb) [file 12896_2016_326_MOESM4_ESM.pdf]

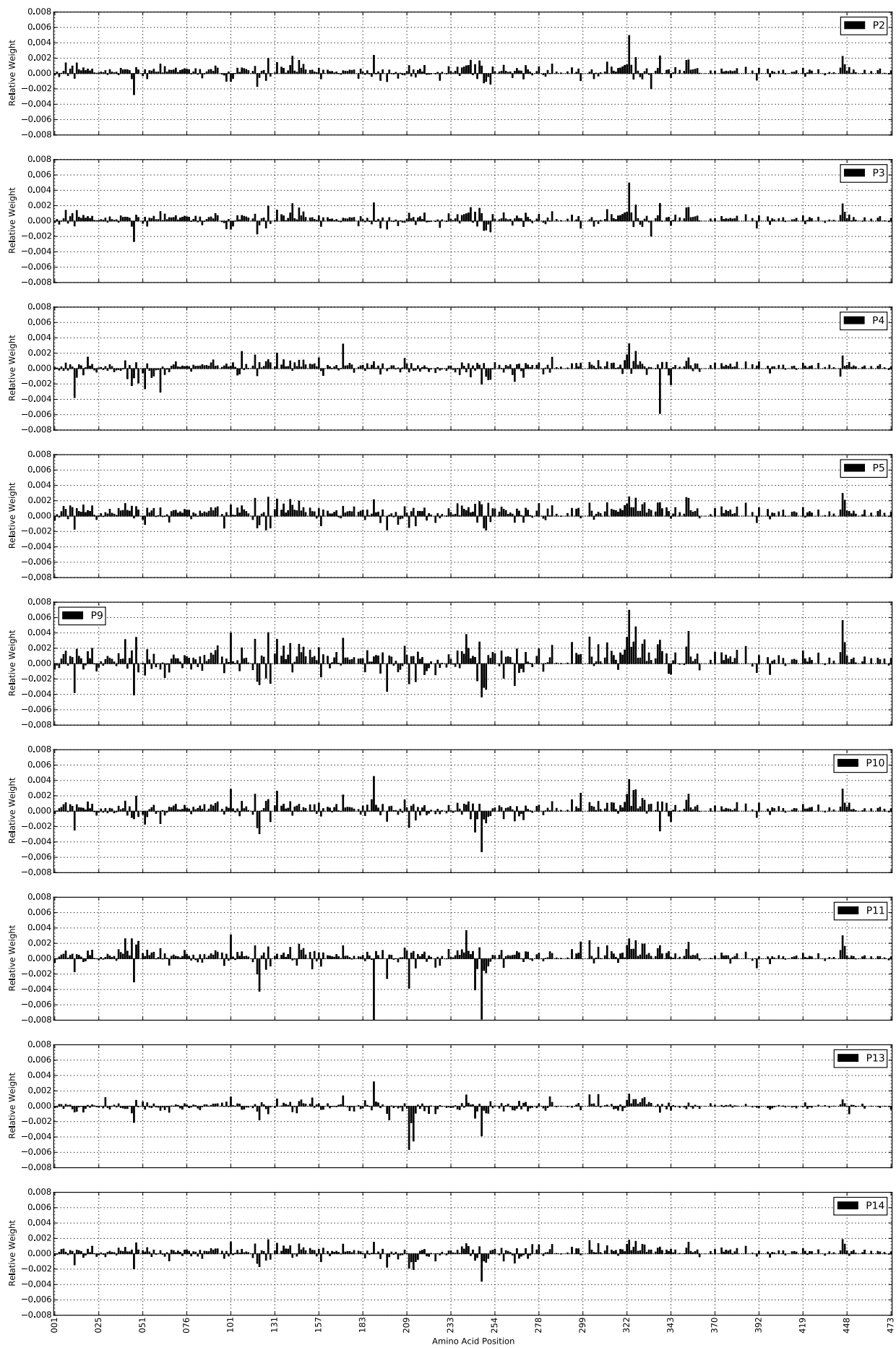

Supplement: Additional file 5: Figure S4. — Linear regression weights on all mutated positions. In case of multiple mutations at a position, the one with the largest effect is shown. Negative values help decreasing the bias while positive values increase the bias. Each plot shows the results of a separate linear regression on a position in the bias curve. (PDF 300 kb) [file 12896_2016_326_MOESM5_ESM.pdf]

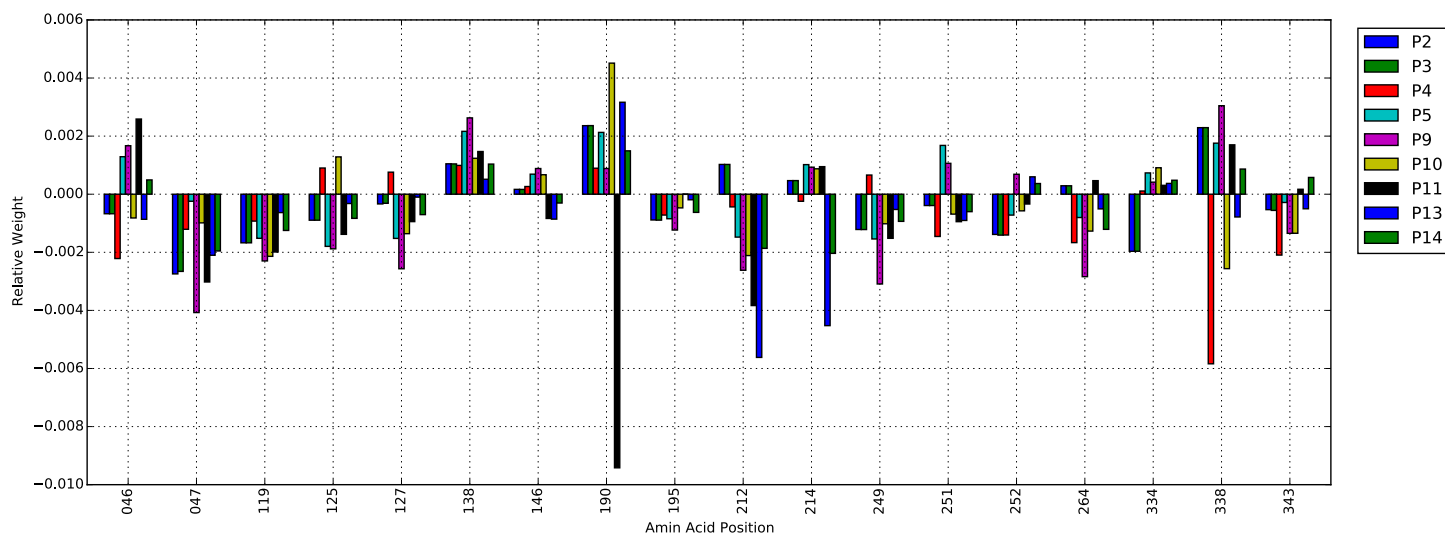

Supplement: Additional file 6: Figure S5. — Linear regression weights on selected amino acids positions. This plot shows the different effect of a mutation on the insertion bias at different positions. (PDF 58 kb) [file 12896_2016_326_MOESM6_ESM.pdf]

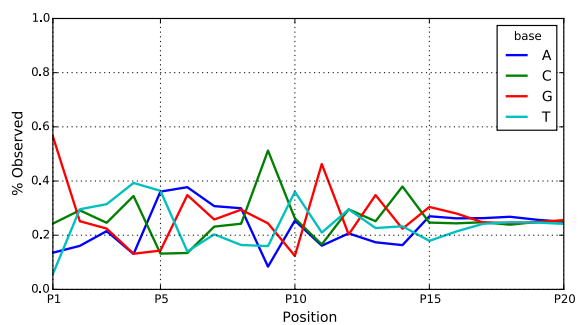

(a)

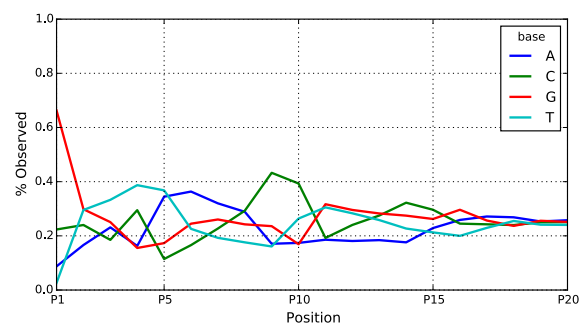

(b)

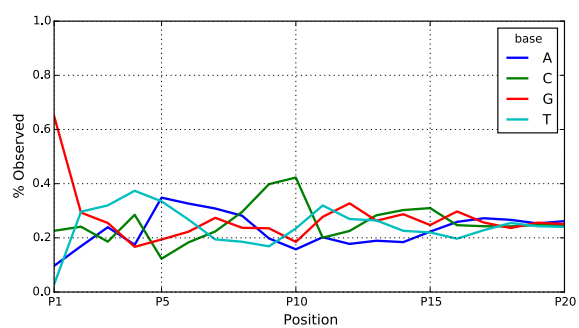

(c)

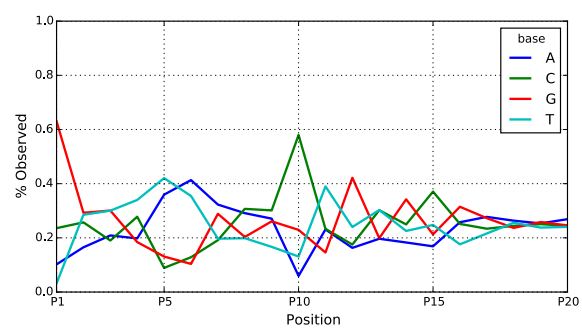

(d)

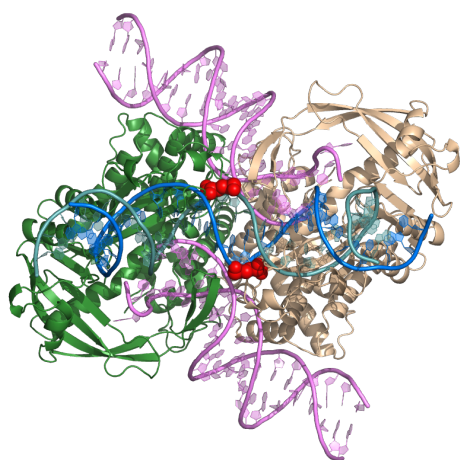

(e)

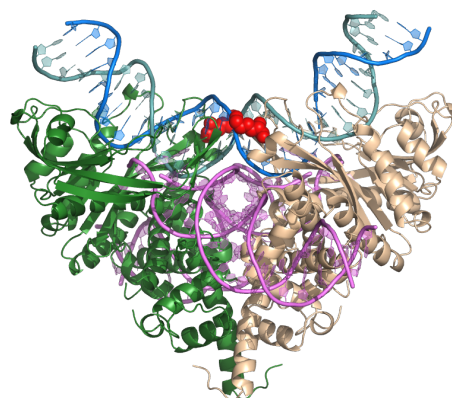

(f)

Supplement: Additional file 7: Figure S6. — Bias plots showing how bulky mutations at K120 distort or shift the symmetry of the graph. (a) NexteraV2 Tn5 (b) K120Y. Symmetry is distorted, resulting from a combination of 9 bp and 10 bp target DNA overlap, with 9 bp dominating (c) K120F. Similar to (b), but 10 bp overlap dominates (d) K120W. Complete switch to 10 bp overlap and the center of symmetry shifts from position 5 to middle of positions 5 and 6 (e) Top view of the Tn5 structure, MEs are shown in magenta color. Target DNA is schematically shown in the figure in shades of blue. K120 is shown in the spherical representation in color red (d) Top view of the structure. (PDF 1226 kb) [file 12896_2016_326_MOESM7_ESM.pdf]
